# Neutral theory and beyond: A systematic review of molecular evolution education

**DOI:** 10.1002/ece3.10365

**Published:** 2023-07-28

**Authors:** Desiree Forsythe, Jeremy L. Hsu

**Affiliations:** ^1^ Grand Challenges Initiative, Schmid College of Science and Technology Chapman University Orange California USA; ^2^ Schmid College of Science and Technology Chapman University Orange California USA

**Keywords:** evolution curriculum, evolution education, molecular evolution

## Abstract

Molecular evolution—including the neutral theory of molecular evolution—is a major sub‐discipline of evolution and is widely taught in undergraduate evolution courses. However, despite its ubiquity, there have not been any previous attempts to compile and review the molecular evolution education literature. Here, we draw upon the framework proposed in a past literature review examining the broader evolution education landscape to conduct a literature review of papers related to molecular evolution education, classifying the contributions of such papers to evolution pedagogy as well as evolution education research. We find that there remains very limited coverage of molecular evolution in the education literature, with existing papers focusing primarily on providing new instructional modules and strategies for teaching molecular evolution. Our work suggests several areas of critical need as well as opportunities to advance evolution education and evolution education research, including compiling instructional goals for the sub‐discipline, developing validated assessments, and investigating student thinking related to molecular evolution. We conclude by providing general strategies, advice, and a novel curricular activity for teaching molecular evolution and the neutral theory of molecular evolution.

## Abstract 2

Molecular evolution is a major sub‐discipline of evolutionary biology and is commonly taught in undergraduate evolution courses. However, despite its ubiquity, there have not been any prior attempts to compile, synthesize, and review the education literature relating to molecular evolution. Here, we provide a literature review on this important field, applying a framework to categorize the available papers in molecular evolution education by their aims and scope, and provide a novel activity and advice for teaching molecular evolution.

## 1 INTRODUCTION

Molecular evolution, or the study of changes in DNA, RNA, and proteins over time, represents a major subfield of evolutionary biology that began to emerge in the early 1900s and rapidly developed in the 1960s (Suárez‐Díaz, 2009). The field has continued to grow and develop with new technological advances, including innovations in genetic and genomic sequencing capabilities, and now encompasses multiple journals, societies, and conferences dedicated to studying molecular evolution (Suárez‐Díaz, 2009; Wolfe & Li, 2003). As the field progresses, there have been recent retrospective reviews examining the current frontiers and lines of research within the discipline, including macromolecular and functional evolution, theoretical population genomics, and beyond (Liberles et al., 2020). These introspective looks at molecular evolution have also highlighted how advances in several subfields, such as population genomics, have become increasingly driven by studies relying on large amounts of molecular data that are enabled by technological advances in sequencing (Bleidorn, 2016; Casillas & Barbadilla, 2017; Goodwin et al., 2016; Liberles et al., 2020).

However, despite the importance of molecular evolution, there have only been a very limited number of studies examining how such concepts are taught and how students learn these concepts in the undergraduate biology classroom, and very few resources published to guide instructors in teaching these principles. This lack of resources is particularly striking given that a study that reviewed syllabi from upper‐division undergraduate evolution courses revealed that nearly 80% of those courses included molecular evolution as one of their main topics (Ziadie & Andrews, 2018), suggesting an urgent need to examine and support molecular evolution education. Here, we provide the first comprehensive examination of molecular evolution education that we are aware of, as well as the first perspectives for instructors on the teaching of molecular evolution and the neutral theory of molecular evolution, a concept critical to molecular evolution. We begin by conducting a literature search of existing published work relating to molecular evolution education, analyzing the results to identify areas of future growth for the evolution education community. We then use this framework to discuss crucial areas of future work to support the teaching of molecular evolution in undergraduate biology classes. We conclude by examining how the neutral theory of molecular evolution is covered in the most common textbooks for undergraduate evolution classes before providing some general recommendations for instructors and a curricular activity that teaches key principles relating to the neutral theory.

## 2 LITERATURE REVIEW FOR MOLECULAR EVOLUTION EDUCATION

### 2.1 Past literature reviews of evolution education reveal very little work pertaining to molecular evolution

We started our literature search by first reviewing the literature for any past attempts to compile or synthesize papers relating to molecular evolution education. We conducted a keyword search using both Google Scholar and the Education Resources Information Center (ERIC), and also searched within several journals that publish work relating to evolution education, such as *Evolution: Education and Outreach*; *CBE‐Life Sciences Education*; and *Ecology and Evolution*, which includes an Academic Practice in Ecology and Evolution section (Moore et al., 2017). The only relevant paper we identified was a systematic review of literature relating to evolution education published between 1990 and 2016 (Ziadie & Andrews, 2018). This review identified over 300 papers published during this period, which were each classified by their subdiscipline of evolution. Fewer than 4% of these papers were related to molecular evolution, placing it as one of the least covered subdisciplines of evolutionary biology in evolution education (Ziadie & Andrews, 2018). In addition, the authors also classified each paper by how they contributed to four commonly recognized categories of pedagogical content knowledge (PCK), or the topic‐specific knowledge that instructors need to effectively communicate and teach concepts related to that topic (Park & Oliver, 2008; Shulman, 1987). Thus, the authors classified each of the papers by if they described a new instructional strategy, examined student thinking, developed new instruments for assessments, or set education goals (Park & Oliver, 2008; Ziadie & Andrews, 2018). Each of the papers identified pertaining to molecular evolution described a new instructional strategy, including new curriculum or modules for teaching different aspects of molecular evolution; none of the papers relating to molecular evolution examined student thinking, developed new instruments, or set educational goals (Ziadie & Andrews, 2018). Taken together, this work suggests that there was a scarcity of work pertaining to molecular evolution education during the years examined, with the limited work available focusing exclusively on curriculum and instructional strategies. In addition, the authors also highlight the need to conduct additional literature reviews on specific sub‐disciplines in evolution, citing how such literature reviews are relatively rare but are likely to have a large impact in supporting instructors who are teaching evolution (Ziadie & Andrews, 2018).

### 2.2 Identifying additional papers relating to molecular evolution education

Given that this previous review had identified molecular evolution education papers published up until October 2016, we focused our search on identifying additional papers published in molecular evolution education from 2016 until February 2023. We then compiled these results to form a database of papers relating to molecular evolution education from 1990 to 2023. For our search, we followed the same procedures outlined in Ziadie and Andrews (2018). We used both Google Scholar and ERIC to conduct a keyword search, using “molecular evolution” and the different terms relating to teaching and learning identified in Ziadie and Andrews (2018), applying the same criteria and process. We only included papers that directly pertained to teaching students about patterns of change in DNA, RNA, or protein, and following the lead of Ziadie and Andrews (2018) did not include papers whose primary coverage was on mechanisms of evolution (e.g., natural selection, mutation, migration, or drift) or population genetics unless the paper explicitly discussed rates of mutation, chromatin and protein evolution, the molecular clock, or the neutral theory of molecular evolution. To ensure full coverage, we searched ERIC for each of these topics as well and used Google Scholar to review all papers that cited any identified works relating to molecular evolution education, reasoning that new papers relating to molecular evolution education would be likely to cite past work in this area. Finally, we also reviewed a published list of concept inventories relating to evolution education (Furrow & Hsu, 2019) to determine if any of these concept inventories focused on molecular evolution.

The authors collaboratively searched for these papers, discussing each one to reach consensus on whether the paper related to molecular evolution and should be included in our analysis. This search led us to identify one additional paper published between 1990 and 2016 that was not included in the database from Ziadie and Andrews (2018). In sum, we identify a total of 26 peer‐reviewed papers relating to molecular evolution education (Table S1), double the number initially identified by Ziadie and Andrews (2018). After identifying these papers, we then classified each of the papers in several ways, with the goal of forming a searchable database of articles relating to molecular evolution education that is helpful for both evolution educators looking to teach molecular evolution as well as evolution education researchers. The categories are as follows:

- *Type of PCK*: First, we identified if each paper presented instructional strategies, examined student thinking, developed new validated assessments, or set educational goals, following the framework used by Ziadie and Andrews (2018). A given paper can fall into one or more of these categories, which align with four commonly agreed‐upon areas of PCK (Park & Oliver, 2008).
- *Type of paper*: Similarly, we categorized each paper as being descriptive (i.e., a paper that describes a new curricular module), empirical (i.e., any paper that systematically gathers data to address a research question), a literature review, or a paper that presented an author's perspective, following the definitions presented by Ziadie and Andrews (2018).
- *Student context*: We determined if each paper examined molecular evolution education in the context of high school biology, undergraduate non‐majors courses, introductory biology, or mid/upper‐division evolution classes. While this article focuses on the teaching of molecular evolution in undergraduate biology classes, we included articles that focus on high school biology given that many of the same concepts and themes may be applicable to non‐majors or introductory biology classes at the undergraduate level, and including such papers provides us a more comprehensive examination of molecular evolution education literature. Some papers provided general advice and strategies without providing a specific student context; these were designated as “general”. Similarly, other papers were not explicit in their intended student demographic. In these cases, we marked any student context suggested by the instructors (e.g., undergraduate versus high school) and then examined the learning objectives and materials provided to reach a consensus for which student level the paper would be most appropriate for. We noted any of these student contexts that were inferred from the activity and were not explicitly described in the manuscript with an asterisk in our table.
- *Length and setting of curricular module*: For papers that presented instructional strategies, we noted the length of the module, classifying if the activity requires one class period or multiple classes. Given the variation in length for multi‐class modules, we also provided additional details for the suggested number of classes required. For those papers describing general advice and strategies rather than a specific module, we denoted this as “general”. Some papers did not provide estimated lengths for their curricular module, and we again drew inferences based on the materials to estimate the amount of instructional time required. Finally, we also noted if the intervention or curricular module was designed for a course that has a lab setting, which typically requires several hours of time in a row and access to either computational or experimental equipment.
- *Coverage of molecular evolution sub‐discipline*: We further classified which sub‐disciplines of molecular evolution that the paper covered. We started by utilizing the list of topics generated by Ziadie and Andrews (2018), which included rate of mutation, chromatin evolution, protein evolution, and molecular clock. We added neutral theory as a sub‐discipline, given its importance to molecular evolution, and iteratively refined this list of sub‐categories as we read through the papers. The final list includes mutations and sequence changes; chromatin evolution; protein evolution; molecular clock; molecular systematics; and neutral theory. Many papers included coverage of topics beyond molecular evolution as well; in these cases, we only classified the relevant molecular evolution sub‐discipline. Similarly, some papers provided general curricular advice for molecular evolution or included coverage spanning across multiple topics relevant to molecular evolution; these were all classified as “general”.

To ensure reliability and trustworthiness of the results, the two authors independently read each of the papers (including the 13 papers originally identified by Ziadie & Andrews, 2018) and iteratively discussed until reaching consensus on each of the categories. Both authors hold graduate degrees in evolutionary biology and have published numerous papers in these areas, regularly teach or have previously taught undergraduate courses that incorporate evolutionary concepts, and are active in biology and evolution education research.

### 2.3 Literature search reveals an increasing, but limited, number of papers relating to molecular evolution education

Our search revealed a total of 26 papers (Table S1), which are sorted in chronological order. We note several implications from our search. First, there remains a highly limited number of papers pertaining to molecular evolution education, despite its importance and widespread coverage in undergraduate evolution courses. While we did not conduct a systematic review of all evolution education papers, we note that there were over 300 articles published between 1990 and 2016 identified by Ziadie and Andrews (2018), with likely a large number of additional papers published since 2016. Thus, the 26 papers we found relating to molecular evolution still represents a small fraction of the overall evolution education body of literature, suggesting that this area of evolution is severely underrepresented in the evolution education literature.

However, we also note that there appears to be an increasing rate of publications relating to molecular evolution education. For instance, Ziadie and Andrews (2018) identified 13 papers relating to this area between 1990 and 2016, a span of 26 years. In contrast, we find an almost equal number of papers relating to molecular evolution education from late 2016 to early 2023, a span of approximately 6 years. While the sample sizes are small, these data suggest that there may be an increasing rate of publications in molecular evolution education. We speculate that such an increase may be driven by the advances in molecular technologies within the last decade, which may lead to renewed focus and attention on molecular evolution education (Casillas & Barbadilla, 2017).

### 2.4 Analysis of molecular evolution education literature reveals several themes and gaps in the literature

We analyzed the body of molecular evolution education literature and identified several themes as well as areas of opportunity for the evolution education community. First, we determined that all the published articles relating to molecular evolution education provide instructional strategies, with papers that share new curriculum and approaches to teaching different facets of molecular evolution. In contrast, there were no papers that addressed any of the other areas of PCK, with zero papers examining student thinking about molecular evolution, establishing validated assessments for molecular evolution, or setting goals for molecular evolution education. In addition, among these papers that provided instructional strategies, nearly 70% (18 of 26) were descriptive, with fewer than one third providing any empirical data designed to answer a research question. The descriptive papers provide general descriptions and information for implementing a curricular module and may include anecdotal data from students and instructors (e.g., comments and feedback on the instruction), but do not include systematic collection of data to assess the efficacy of the module. However, we note that the relative number of instructional papers with empirical data may be increasing: only two papers that Ziadie and Andrews (2018) identified that were published between 1990 and 2016 (a period of 26 years) included empirical data, while we identified four additional empirical papers published between 2016 and 2023 (a period of approximately 6 years), in addition to a paper from 2012 not previously included in Ziadie and Andrews (2018).

We also identify that the published instructional materials are designed for a range of student levels. Half of the papers were designed for undergraduate students in upper‐division biology classes (including a paper that described a curricular module designed for both introductory and upper‐level students), followed by approximately a third (34.6%) of approaches designed for high school students. In contrast, fewer than one fifth (19.2%) of instructional approaches were geared for introductory biology students, and only three papers described applying the module for undergraduate non‐majors biology courses. Similarly, we find that the published approaches are designed for a diversity of lengths and formats. For instance, a little over half of published approaches (54%) require multiple class sessions, while approximately 40% are designed for a single class (with the remaining papers providing general strategies rather than specific curriculum). In addition, while the majority of papers describe modules that can be implemented in traditional classrooms, several require computational or molecular labs to support the activity.

Finally, we identify uneven coverage of topics in molecular evolution. For instance, we find that some topics, such as protein evolution, mutations and sequence changes, and the molecular clock, were each covered by multiple papers' curricular modules. In contrast, other topics, such as chromatin evolution, RNA evolution, and molecular systematics, only had one paper each providing curriculum in those areas. In addition, we note the major challenge of classifying these instructional papers by their molecular evolution topic. There is no clear, consensus list of learning objectives or competencies related to molecular evolution, nor is there a widely agreed upon list of sub‐disciplines within molecular evolution. In addition, many of the papers included in our review did not list their learning objectives, making it at times challenging to infer the primary goals of the curriculum, and multiple papers described activities that focused upon concepts outside molecular evolution but included some coverage of molecular evolution concepts, again presenting challenges in classifying the molecular evolution topics. Given these challenges, we acknowledge that different evolutionary biologists may bin the coverage areas of each paper differently. We note that our goal of classifying these papers is not to provide a comprehensive list of topics that each paper covers, but instead to determine if there is even coverage of molecular evolution topics across the literature, and to provide a general framework that instructors can use as a starting place for identifying and choosing papers with relevant activities for their courses.

Interestingly, we find that none of the papers focused on teaching the neutral theory, indicating that there are currently no published, peer‐reviewed resources for teaching neutral theory or studying how students think about and conceptualize such principles. Only two articles explicitly mention the neutral theory. The first, a lab activity where students build a phylogenetic tree after extracting and sequencing their own DNA, primarily focuses on other evolutionary principles, such as phylogenetics and population genetics (Maroja & Wilder, 2012). The activity does introduce the molecular clock, which was proposed prior to the neutral theory but is now widely accepted as built upon the foundations of neutral theory (Gojobori et al., 1990). However, the activity does not provide significant coverage to any of the neutral theory's core ideas surrounding neutral evolution. The second article mentions the neutral theory in their list of topics covered in their course, but does not provide any instruction, curricular materials, or advice for teaching the neutral theory (Yoshida & Page, 2022). Similarly, two other published articles discuss teaching approaches that involve neutral mutations or some genes evolving neutrally, but again do not explicitly define or characterize neutral theory (Miralles et al., 2013; Shumate & Windsor, 2010).

## 3 IMPLICATIONS FOR EVOLUTION EDUCATION AND BIOLOGY EDUCATION RESEARCH COMMUNITIES

Based on this literature review, we highlight the following implications for the evolution education and biology education research communities. First, we call on these communities to examine the teaching and learning of molecular evolution more in depth, including investigating how students conceptualize molecular evolution, exploring if there are common challenges or misconceptions students have when learning key concepts related to molecular evolution, and developing validated instruments to measure student learning in these areas. There were no papers in any of these areas, and our examination of concept inventories related to evolution also did not identify any papers related to molecular evolution. This lack of work severely limits the development of evidence‐based pedagogical approaches. For instance, studies that examine how students conceptualize different topics within molecular evolution can provide insight into how students think about these topics, and what misconceptions they may hold (Leonard et al., 2014). These insights can inform the development of curriculum and pedagogical interventions to address such misconceptions. Similarly, the development of validated instruments to assess student learning can be invaluable for both instructors and the evolution education research community. For example, validated instruments can be used to better assess the efficacy of different instructional approaches and can be used by instructors to identify possible areas to teach in a course (Furrow & Hsu, 2019). Other instruments can also help identify how students think about molecular evolution, thus informing research into how students build mental models when learning molecular evolution (Smith & Tanner, 2010; Ziadie & Andrews, 2018). We also highlight the significant proportion of instructional activities aimed at high school students, suggesting that there may be opportunities to develop such instruments and investigate student thinking about molecular evolution in both high school and undergraduate introductory biology courses.

In addition, there is a need to identify “big ideas” and core concepts of molecular evolution for the undergraduate classroom. The literature review identified that the published instructional strategies designed for both high school and undergraduate courses covered a diverse set of molecular evolution principles. However, it remains unclear (1) which concepts and competencies in molecular evolution are most frequently taught in undergraduate biology and evolution courses (and to what extent these topics are covered or previewed in high school biology courses) and (2) which concepts in molecular evolution most instructors, faculty, and evolutionary biologists consider as the most important. There has been a recent effort in other subfields of evolution, such as evolutionary medicine, to establish a list of “big ideas” or “core concepts” (Grunspan et al., 2018), and developing detailed learning objectives can help improve student learning, assessment, and instruction (Clark & Hsu, 2023; Orr et al., 2022a, 2022b). This approach of iteratively surveying experts to identify the most important ideas for students to learn has also been conducted in physiology and is highlighted in the seminal *Vision & Change* report for transforming undergraduate biology education (American Association for the Advancement of Science (AAAS), 2011; Hsu & Halpin, 2022; Michael et al., 2009; Michael & McFarland, 2011). Indeed, *Vision & Change* advocates for establishing core concepts for each discipline to guide instructors on what concepts to teach and promotes instructors introducing concepts through a broader conceptual framework rather than as isolated facts (American Association for the Advancement of Science (AAAS), 2011; Grunspan et al., 2018). In addition, developing core concepts for a discipline or subdiscipline can facilitate the development of new curriculum aligned with such core concepts as well as introspective studies that examine the teaching of such core concepts in the undergraduate classroom (Hsu & Halpin, 2022). Thus, establishing a set of big ideas and core principles for molecular evolution will benefit instructors and the broader evolution education community.

Finally, we urge these communities to develop more resources relating to molecular evolution. Our work finds that there remains an extremely limited number of papers and studies relating to molecular evolution education. Given how commonly taught molecular evolution is at the undergraduate level and the importance of molecular evolution in evolutionary biology, there is an urgent need to create more resources that can facilitate evidence‐based teaching of molecular evolution. Additional curricular modules, particularly those that present concepts in areas that currently have no coverage (e.g., neutral theory) or limited coverage (e.g., RNA and chromatin evolution) can be particularly impactful for instructors looking to incorporate additional coverage of molecular evolution in their courses. Similarly, given that the majority of published approaches are designed for upper‐division undergraduate courses or high school courses, there is a major need to develop additional modules specifically geared towards introductory biology and non‐majors courses. In addition, we note that there is an opportunity to assess the effectiveness of currently published modules, most of which were published as descriptive papers and do not provide any formal assessment data. We encourage instructors who implement such curricular modules or strategies to consider systematically gathering and sharing assessment data and other information that can inform how students are thinking about and conceptualizing molecular evolution concepts. Such instructors may wish to consult some of the published resources that are designed to guide instructors who are new to assessment and discipline‐based education research (e.g., Cole & Bunce, 2014; Dancy & Beichner, 2002; St John, 2016). We also note that the development of such resources and curriculum can be informed by studies that explore how students think about molecular evolution. For instance, research that identifies key student misconceptions in a given area of molecular evolution can lead to the development and assessment of curriculum designed to elicit and counter those misconceptions. Similarly, work that identifies core concepts in molecular biology can be used as a framework to organize and classify teaching resources and curriculum relating to molecular biology and can also be used to identify areas of low or missing coverage as more resources are developed.

## 4 EXAMINING DIFFERING APPROACHES FOR TEACHING MOLECULAR EVOLUTION: NEUTRAL THEORY AS A CASE STUDY

Our literature review of molecular evolution education revealed that there is a paucity of literature examining how students learn molecular evolution or how molecular evolution is taught in undergraduate biology classrooms. We conclude this paper by providing a case study that examines the teaching of the neutral theory of molecular evolution and offering recommendations for instructors on the teaching of the neutral theory and molecular evolution, drawing upon our own experiences and the framework identified by our literature review. We focus on neutral theory for both theoretical and practical reasons. First, the neutral theory is considered to be of high importance in molecular evolution. The theory, first proposed in 1968 by Motoo Kimura, has emerged in the subsequent decades as a hotly debated yet critical perspective on understanding what forces drive molecular evolution (Jukes & Kimura, 1984; Kimura, 1968, 1983). The theory, which highlights the importance of genetic drift in driving neutral evolution (Jensen et al., 2019; Leigh, 2007), has led to continual scientific discourse over its applicability (Jensen et al., 2019; Kern & Hahn, 2018), and there have been refinements and extensions of the neutral theory proposed. For example, the nearly neutral theory was proposed in 1973, revising the neutral theory by proposing that many mutations are slightly deleterious but may still act similarly to completely neutral mutations and become fixed in a population (Casillas & Barbadilla, 2017; Ohta, 1973). In addition, the neutral theory has been used to explain the patterns behind the molecular clock—the observation that many organisms exhibit constant rates of accumulated change in certain genes or molecules (Casillas & Barbadilla, 2017; Kimura, 1987; Thorpe, 1982)—which was first proposed prior to the neutral theory (Morgan, 1998; Zuckerkandl & Pauling, 1965). Given the wide application of the neutral theory, it has become a “unifying frame” for many molecular evolution studies and is now regarded as a “guiding principle for studying evolutionary genomics” (Leigh, 2007; Nei, 2005; Nei et al., 2010), indicating that it is of critical importance to support the teaching of neutral theory in undergraduate biology. Second, we identified that there were no previously published approaches addressing neutral theory in our literature review, suggesting that focusing on this concept as a case study could provide a valuable resource for instructors aiming to teach neutral theory. Finally, we focus on the neutral theory for practical reasons as well, given that we have developed and taught an instructional activity for teaching principles related to the neutral theory. These experiences allow us to provide suggestions and recommendations for instructors aiming to teach the neutral theory.

We provide here a summary of the main concepts of neutral theory, but guide the reader to other resources and reviews (e.g., Casillas & Barbadilla, 2017; Jensen et al., 2019) for more detailed information on the neutral theory. In brief, neutral theory states that drift is the predominant force acting on new mutations that remain in the population, given that positive selection is extremely rare and that purifying selection removes deleterious mutations (Kimura, 1983). Given this, neutral theory predicts that the rate of substitution of new mutations in a population is equal to the rate of neutral mutations in an individual, a mathematical calculation that has been empirically observed (Jukes & Kimura, 1984).

### 4.1 Evolution textbooks provide differing approaches for introducing the neutral theory

We first examine several key textbooks for evolutionary biology to characterize how these texts introduce the neutral theory. Textbooks serve as a key source of information for instructors, often informing the choice of topics in a course and also commonly influencing how instructors present certain concepts (Abd‐El‐Khalick et al., 2008; Hsu & Halpin, 2022; Valverde et al., 2002). Thus, examining how textbooks present the neutral theory can provide insight into how instructors may be teaching the neutral theory in undergraduate biology courses.

We examined four textbooks that have been identified as the most used texts in undergraduate mid‐ and upper‐level evolution courses in the United States: Herron and Freeman's *Evolutionary Analysis* (Herron & Freeman, 2007), Futuyma and Kirkpatrick's *Evolution* (Futuyma & Kirkpatrick, 2013), Bergstrom and Dugatkin's *Evolution* (Bergstrom & Dugatkin, 2012), and Zimmer and Emlen's *Evolution: Making Sense of Life* (Zimmer & Emlen, 2013). Together, these texts are used in over 90% of evolution courses based on a survey of over 200 evolution courses (Fuselier et al., 2016). We started by identifying the relevant chapters for molecular evolution and the neutral theory by consulting both the table of contents and the indices for each of the textbooks. Next, each author independently read the relevant sections before discussing with each other. These discussions allowed us to classify the approaches used for teaching neutral theory in three separate ways:

1. *The context of where the neutral theory is introduced in the textbook* (i.e., the main topics covered in the relevant chapter). We examine the context to better situate how neutral theory is presented and what other concepts are presented alongside the neutral theory.
2. *How the textbook connected the molecular clock to the neutral theory* (i.e., if the molecular clock was discussed prior to or after the discussion of the neutral theory and if the text made explicit connections between these principles). We examine the placement of teaching about the molecular clock to the neutral theory given that we identified several published resources for teaching about the molecular clock but none on the neutral theory, and that the neutral theory is often used to explain the constant rates of change in molecular clocks (Bromham & Penny, 2003; Kimura, 1987; Takahata, 1996).
3. *If the text discussed how the rate of neutral evolution is equal to the rate of neutral mutation under the neutral theory, regardless of population size*. We examine if the text discusses how the rate of neutral evolution is equal to the rate of neutral mutation, independent of the population size, given that this equivalence has been described as “one of the most elegant mathematical expressions of science” (Casillas & Barbadilla, 2017) and that our activity focuses on guiding students to discover this concept.

We find significant variation in how textbooks approach the teaching of the neutral theory of molecular evolution (Table 1), suggesting that there is also likely widespread diversity in how instructors approach these concepts and the level of coverage across undergraduate evolution courses. For instance, we find variation in where the neutral theory is discussed: while three of the four texts introduce the neutral theory in the context of genetic drift as an evolutionary mechanism, the fourth text integrates the discussion of neutral theory within a chapter that instead focuses on coalescent theory and molecular phylogenetics, rather than on genetic drift as an evolutionary mechanism. Similarly, we see variation in how these texts connect the neutral theory and molecular clock. While each of the texts links these two concepts, some provide more explicit connections than others, and some texts introduce the molecular clock prior to discussing neutral theory. In addition, we see variation in the extent that these textbooks discuss the concept that the rate of neutral evolution is equal to the rate of neutral mutation under the neutral theory. One of the four texts did not cover this principle, while a second text mentioned this expression but did not provide any mathematical justification or derivation of this principle (we guide the reader to Casillas & Barbadilla, 2017 for a brief overview of this mathematical justification). We also note that none of the texts guide students to discover this concept themselves, with the two texts that offer a mathematical justification relying on mathematical calculations of this principle using abstract variables (e.g., *μ* for mutation rate and *N* for population size). This approach may not be the best for supporting student learning, given that past studies have demonstrated that students may have more challenges comprehending and interpreting symbolic representations of numbers (Jack & Thompson, 2008; Koedinger & Nathan, 2004). Finally, we note differences in the length and depth of coverage of the neutral theory. While we did not systematically analyze the level of coverage given that different textbooks have different formats, font sizes, and page counts, we note that some textbooks, like Zimmer and Emlen (2013), dedicate only a brief paragraph to the neutral theory, while others, such as Bergstrom and Dugatkin (2012), provide a more comprehensive discussion that span multiple pages. Future work is needed to examine how these differing approaches for presenting the neutral theory influence instructional decisions regarding how these concepts are taught in undergraduate biology courses, as well as the impact on students' conceptual thinking on the neutral theory.

**TABLE 1:** Snapshot of how neutral theory is approached in the most commonly used evolution textbooks for undergraduate biology courses.

Textbook	Context of discussion of neutral theory	Connection to molecular clock	Discussion of the rate of neutral evolution equal to the rate of neutral mutation
*Evolutionary Analysis*, Herron and Freeman	Placed within chapter on migration, drift, and non‐random mating	Briefly introduces molecular clock in earlier chapter; connects neutral theory to molecular clock, then includes further discussion of molecular clock in a later chapter	Discussed and includes mathematical justification
*Evolution*, Futuyma and Kirkpatrick	Placed within chapter on genetic drift; discussed again briefly in chapter on gene and genome evolution	Introduces molecular clock prior to neutral theory, then contextualizes neutral theory as an explanation for the molecular clock	Not discussed
*Evolution*, Bergstrom and Dugatkin	Placed within chapter on “Evolution in finite populations”, which begins with genetic drift	Discusses molecular clock after introducing neutral theory and various tests for selection (e.g., dN/dS and McDonald‐Kreitman tests)	Discussed and includes mathematical justification
*Evolution: Making Sense of Life*, Zimmer and Emlen	Placed within chapter on “The History in Our Genes,” which discusses coalescent theory and molecular phylogenetics prior to introducing neutral theory	Discusses molecular clock after introducing neutral theory	Discussed but without any mathematical justification

### 4.2 A sample curricular activity for teaching neutral theory

Given these disparate approaches for teaching neutral theory, we offer here a novel, inquiry‐based instructional activity for guiding students to think more concretely and make inferences about a core principle of the neutral theory that does not rely on symbolic representations of variables and instead challenges students to think critically about simulated populations that include different rates of mutations and population sizes (see supplemental files for the student handout, instructor key, and instructor guide).

This activity is primarily geared for mid‐ to upper‐level undergraduate evolution courses and is designed to be completed in one class session. There are several learning objectives; by the end of the module, students should be able to:

- Mathematically calculate the rate of neutral evolution in different populations
- Explain why the rate of neutral evolution is equal to the rate of neutral mutation under the neutral theory
- Draw inferences about how a constant rate of neutral evolution can inform the molecular clock

Prior to this lesson, students should be familiar with the evolutionary mechanisms, including mutations, migration, drift, and selection, and should recognize that the probability of fixation for a given allele is equal to its frequency in the population if drift is the only force at play. In addition, instructors should introduce the key ideas of the neutral theory prior to this activity and provide context for why it is important to mathematically calculate the rate of neutral evolution in various populations if the main principles of the neutral theory hold true. Casillas and Barbadilla (2017) provide a comprehensive summary of the main concepts of the neutral theory, and instructors may wish to review Box 1 in that article, which provides a summary of main points related to the neutral theory, prior to implementing this activity.

The activity is set up around students working with two sample populations with the same neutral mutation rate, but different population sizes. Students are challenged to think critically and calculate the number of new neutral mutations, the probability of each new mutation reaching fixation under neutrality, and the expected number of neutral mutations reaching fixation. Students are guided to reach the conclusions that population size has no impact on the rate of neutral evolution, and to discover that the rate of neutral evolution is always equal to the rate of neutral mutation. This activity is thus organized as an inquiry‐based activity where students are asking and addressing key questions pertaining to the neutral theory and also developing quantitative reasoning skills, a core competency identified as important in many national calls, such as *Vision & Change* (American Association for the Advancement of Science (AAAS), 2011). In addition, we note that this activity presents a different approach than how this concept is addressed in the most common evolution textbooks, none of which guide students to make this discovery by themselves. Instead, here we adopt an inquiry‐based learning approach, where students are challenged to think critically and draw inferences from the scenarios and provided data. While we have not gathered any assessment data on our activity, inquiry‐based learning has been demonstrated to be more beneficial to student learning than didactic approaches where the instructor provides the key concept (Abdi, 2014; Lord & Orkwiszewski, 2006). This difference in learning is likely due to the fact that inquiry‐based approaches mirror the scientific process, providing additional opportunities for students to engage critically with puzzling observations (Gehring & Eastman, 2008; Harlen, 2013; Roehrig et al., 2012).

## 5 LIMITATIONS

We recognize several limitations of our work. First, while we made every effort to be comprehensive in our literature review, we acknowledge that there is the possibility that additional literature exists that is relevant to molecular evolution but not found through our keyword search. In addition, our search followed the approach of the systematic review reported by Ziadie and Andrews (2018) and used explicit, pre‐defined criteria. However, past meta‐analyses of literature have identified that there is no consensus for what a systematic review is, with a wide diversity of definitions and elements in the literature and large variation in how systematic reviews are conducted within education research (Bearman et al., 2012; Krnic Martinic et al., 2019). We acknowledge that our search does not encompass all elements that have been applied to systematic reviews in other contexts, such as assessing the quality of studies or the risk of publication biases (Kim et al., 2015). In addition, we echo the inherent limitations that Ziadie and Andrews (2018) highlight of literature reviews, where reviews may become out of date as additional papers related to molecular evolution are published. We also note how there are many curriculum or education research papers investigating topics adjacent to molecular evolution, that is, topics like mutation and genetic drift that are fundamental to studying molecular evolution, that were not classified as part of molecular evolution in our framework and thus not included in our review. However, we highlight how the instructor guide provides a short summary of this body of literature to better guide instructors. Finally, we note that there were subjective elements of our classification scheme. For instance, we chose several broad, higher‐level categories when classifying each paper's sub‐discipline of molecular evolution, but it is possible that others may see more value in providing more specific, narrow sub‐disciplines. Despite these limitations, our work provides the first literature review on molecular evolution education that we are aware of and provides a valuable framework for both instructors and education researchers to use in future work examining molecular evolution education.

## AUTHOR CONTRIBUTIONS

**Desiree Forsythe:** Conceptualization (equal); data curation (equal); writing – original draft (equal); writing – review and editing (equal). **Jeremy L. Hsu:** Conceptualization (equal); data curation (equal); writing – original draft (equal); writing – review and editing (equal).

## CONFLICT OF INTEREST STATEMENT

The authors declare no conflicts of interest.

## Supporting information


Table S1.

Appendix S1.

## Data Availability

The data that supports the findings of this study are available in the supplementary material of this article.
