# Supplementary material for: Neutral theory and beyond: A systematic review of molecular evolution education
Source: Ecol Evol. 2023 Jul 28;13(8):e10365. doi: 10.1002/ece3.10365 (PMC10375367; doi:10.1002/ece3.10365)
Supplement: Supplementary file 2 — Appendix S1. [file ECE3-13-e10365-s002.docx]

**Supporting information for Forsythe and Hsu**

**Table of contents**

1. Instructor guide - pages 3-9
2. Student handout – pages 10-13
3. Instructor key – pages 14-17
4. References – pages 18-22

**Instructor guide**

The neutral theory of molecular evolution is a key concept in evolutionary biology often taught in undergraduate evolution courses. Under the neutral theory, which is covered in each of the four textbooks that are the most used textbooks in undergraduate evolution classes, beneficial mutations are very rare in a population. Instead, purifying selection and random genetic drift are the main evolutionary forces acting upon mutations. Given that deleterious mutations will be removed from the population by purifying selection, this means that changes in allele frequency of the remaining mutations will be driven by drift. Thus, the neutral theory posits that drift – and not selection – is the driving force of divergence between organisms at the molecular level.

This activity is designed for mid- and upper-level undergraduate evolution classes, though this instructor guide provides suggestions and recommendations for adjusting the activity for introductory level courses. The activity is designed to be completed in one 50-minute class period, though can be extended to longer durations as well (see section below for suggested adjustments). In addition, the activity can be deployed in classes with a range of sizes.

There are three learning objectives for the activity. At the end of the module, students should be able to:

- Mathematically calculate the rate of neutral evolution in different populations
- Explain why the rate of neutral evolution is equal to the rate of neutral mutation under the neutral theory
- Draw inferences about how a constant rate of neutral evolution can inform the molecular clock

*Prerequisite knowledge needed for activity*

This activity is designed to be implemented after instructors have finished teaching about evolutionary mechanisms, including both selection and random genetic drift. While it is beyond the scope of this activity to teach about those mechanisms, we provide here a summary of useful resources to guide the instructor in thinking about their teaching of selection and drift. First, we encourage instructors to review concept inventories for both selection and drift. Concept inventories are assessments, usually multiple choice, that have been rigorously developed and have evidence of their validity and reliability. For instance, concept inventories often start with interviews and surveys of content experts to establish what the main concepts are relating to a given topic, and then usually involve iterative development of questions that assess student conceptual understanding of the given topic. Questions are continually refined during the development process to ensure clarity and consistency. Given this rigorous process, instructors can use concept inventories to identify main concepts and establish learning objectives relating to evolutionary mechanisms, recognize key misconceptions students might have about the concept, and assess student learning. We highlight an evidence-based guide for instructors on how to use concept inventories relating to evolution that may be useful for instructors (Furrow & Hsu, 2019). This publication also compiles the list of all available concept inventories relating to evolution, and instructors may wish to review the Genetic Drift Concept Inventory (Price et al., 2014) and the numerous concept inventories relating to selection (e.g., Anderson, Fisher, & Norman, 2002; Kalinowski, Leonard, & Taper, 2016; Nehm, Beggrow, Opfer, & Ha, 2012; Nehm & Schonfeld, 2008).

Second, we highlight that – unlike molecular evolution – there is a plethora of biology education research that examines students’ learning of natural selection and genetic drift that instructors may wish to review to guide their teaching. For instance, there is work examining undergraduate students’ knowledge of and misconceptions of natural selection (Ferrari & Chi, 1998; Greene Jr., 1990; Gregory, 2009; Nehm & Reilly, 2007) and drift (T. M. Andrews et al., 2012), exploring their patterns of reasoning regarding selection and how they connect principles of genetics to selection (Baumgartner & Duncan, 2009; Lawson & Thompson, 1988; Settlage Jr., 1994; Tibell & Harms, 2017), and investigating students’ conceptions when learning selection and drift together (Beggrow & Nehm, 2012). There are similarly many published activities for undergraduate biology classes designed to teach about these concepts and counter misconceptions, many with assessment data showing efficacy of the activity (e.g., Abraham et al., 2009; Tessa M. Andrews, Kalinowski, & Leonard, 2011; Hsu, Imad, & Wilson, 2021; Kalinowski, Andrews, Leonard, & Snodgrass, 2012; Russo & Voloch, 2012). We encourage instructors to review the framework and corresponding list of literature pertaining to evolution education compiled by Ziadie & Andrews (2018), who classify each paper by their content (e.g., selection, drift, population genetics, etc.) and how each paper advances evolution education (e.g., providing a new curriculum, examining student thinking, etc.).

Finally, this activity builds upon several concepts that instructors will need to introduce prior to implementing this activity. For instance, students should be able to calculate allelic frequencies in a population and recognize that the probability of an allele reaching fixation is equal to its frequency in the population if drift is the only force acting upon the allele. Similarly, instructors will need to convey that under the neutral theory that beneficial mutations are exceedingly rare and that deleterious mutations are removed by purifying selection, meaning that drift will be the only force acting upon variation that remains in a population.

*Recommended structure of activity*

This activity is designed to be completed in small groups of two-three students each. Instructors can either print the handouts (see student handout below) or post the handouts on their learning management system. The activity and handout are divided into several sections (see supplemental figure 1 for an overview of the activity, including recommended timing for each section); the student handout and instructor answer key are included later in this guide. First, the opening paragraph of the handout introduces variation in the alkaline phosphatase (*ALP*) gene in humans and provides a short sequence for alleles A1 and A2. The sequences are different by one nucleotide, and instructors can reinforce that alleles are, by definition, variants of a gene. Instructors may wish to highlight these ideas to the class prior to letting them work on the activity. Next, instructors are encouraged to have students work together in groups for 10 minutes on the handout, before bringing the whole class together to discuss the first set of questions (questions 1-8).


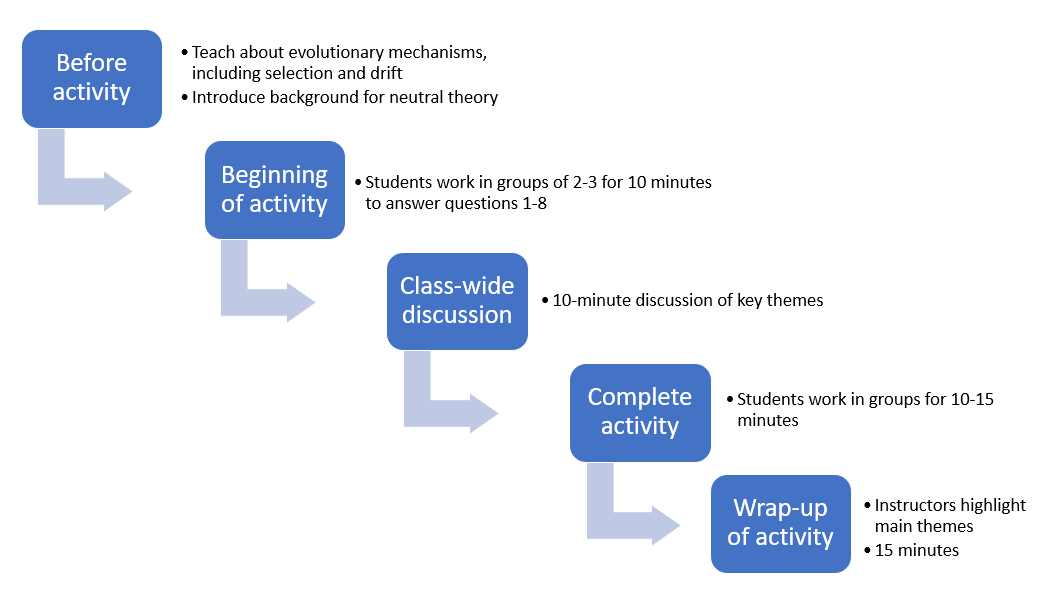


**Supplemental figure 1.** Timeline of activity

These opening questions first guide students to calculate the probability of an allele reaching fixation in the population if drift is the only force acting upon that allele (question 1); instructors can remind students that this probability will be equal to the frequency of the allele in the population. Next, students are asked to think critically about new mutations occurring, which creates new alleles. Students often have trouble conceptually linking a mutation to the creation of a new allele (Smith, Wood, & Knight, 2008), and instructors may wish to reinforce the concept that mutations in a gene, by definition, create new variants of that gene and thus a new allele for the gene. Similarly, students often have trouble conceptually understanding what a mutation is due to the different meanings of the term, which can refer to both the *process* of a change in DNA (i.e., the phenomenon of DNA changing) as well as the resulting *product* (i.e., the altered DNA) (Zhao & Schuchardt, 2019). Instructors may thus wish to explicitly highlight this distinction between process and product and clarify to students what they are referring to each time the word ‘mutation’ is used. For example, when the handout discusses rates of mutation, this is referring to the *process* of DNA changing (i.e., the frequency at which DNA is altered). In contrast, when the handout discusses the probability of a mutation reaching fixation, it is referring to the *product* (i.e., the probability of the resulting allele created by the change in DNA reaching fixation). Instructors should therefore be cognizant of these distinctions and clarify their usage of the term whenever they use the term in their teaching (Zhao & Schuchardt, 2019).

The next questions in this set challenge students to think critically about both the number of mutations expected in the population (question 2) and then what may happen to each mutation once it has occurred (questions 3, 4, and 5). Students will be applying quantitative reasoning skills to calculate the number of expected mutations each generation when given a mutation rate, and to calculate the rate of neutral mutation (assuming that half of the mutations are under purifying selection and the other half are evolving neutrally). Students will then infer that the probability of each neutral mutation reaching fixation is equal to its frequency, which will be 1 divided by the total number of alleles in the population. Given that humans are diploid, the total number of alleles in the population will be two times the number of individuals. Instructors should note that students may become confused thinking about the number of alleles in a population (i.e., the size of the gene pool) and the number of distinct alleles in a population (i.e., the number of different variants of the gene in the population) and may conflate these two. Thus, instructors may wish to directly discuss these differences and highlight why the number of different variants of a gene does not impact the frequency of a given allele and thus does not influence the probability of fixation for that allele (assuming drift is the only force acting on that allele).

Questions 6 and 7 guide students to think about how many of the neutral mutations they expect to reach fixation and to compare this value to the rate of neutral mutation. Students should recognize that these values are equal. Question 8 challenges students to work through the same scenario, but with a different rate of mutation; despite this change, students should still identify that the number of neutral mutations they expect to reach fixation is equal to the rate of neutral mutation. Instructors are encouraged to check on students’ progress during these 10 minutes and to help provide clarification to students as needed; if a class has teaching assistants or learning assistants, it may be helpful for them to circulate among the groups as well. After students are done working on this first set of questions, instructors can bring the class together, ask for volunteers to share their responses, and then discuss the key themes. Instructors are encouraged to work through the mathematical problems on the whiteboard and highlight the major theme that students inferred: that the rate of neutral evolution is the same as the rate of neutral mutation in a population.

Following this class-wide discussion, which should take approximately 10 minutes, instructors can ask students to return to the handout and complete questions 9-17 (see supplemental figure 1). This should take students another 10-15 minutes to complete. By asking students to think through the rate of neutral evolution in populations of different sizes, questions 9-15 challenge students to draw the inference that the rate of neutral evolution will always be equal to the rate of neutral mutation independent of population size. Finally, questions 16 and 17 provide students a chance to connect these ideas with the molecular clock, which instructors can then introduce after this activity. Instructors should then finish the class by asking groups to share their inferences and reasoning, and recapping the main themes from these questions, which should take approximately 15 minutes.

*Potential modifications and assessments for activity*

There are several potential modifications for this activity. First, instructors can utilize polling software (e.g., TopHat, PollEV, etc.) to gather responses from each group for certain questions. This may be particularly useful in large classes, where instructors may not be able to circulate to most groups during the groupwork. For instance, the questions that ask students to calculate a numerical response could become multiple choice questions, where the instructors include both the correct response and several incorrect responses as choices. Alternatively, most of these platforms allow instructors to collect free responses, thus permitting students to type in a fraction as a response. Instructors can then view these responses to gauge the classes’ progress and level of understanding and adjust the teaching accordingly. Similarly, instructors can rely on editable sites (e.g., Google Documents), where instructors ask one student in each group to anonymously type in their response on a document. This method is particularly impactful for questions that ask for more substantial explanations (e.g., questions 15 and 17) since instructors would be able to quickly skim through responses and get a sense of student understanding and could also share students’ responses with the class, allowing students to see how some of their peers reasoned through these problems.

This activity can also be modified for different class levels. For instance, while the activity is designed for a mid- to upper-level evolution class, instructors of introductory biology who wish to teach some principles of neutral theory can deploy parts of the activity. For example, instructors in introductory biology could have students only complete the first set of questions (question 1-8), which introduces the idea that the rate of neutral evolution is equal to the rate of neutral mutation. While students would not have the chance to explore the impacts (or lack thereof) of changing population size, students would still be able to think critically about the influence of drift in driving certain mutations to fixation and still learn about a key principle of the neutral theory. Similarly, instructors of introductory biology could directly provide the allele frequencies in a population for question 1 rather than having students calculate the allele frequency if this is a skill that they do not wish to cover in introductory biology or if they are short on time for the activity. Instructors of more advanced classes can also extend this activity by building in questions that challenge students to think critically about what would happen if the percentage of mutations that are evolving neutrally changes. This activity asks students to assume that half of the mutations are under strong purifying selection and that half are neutral, but these numbers can be varied to challenge students’ thinking under different scenarios, and instructors can highlight how neutral mutation rates can be highly variable (even across genes and organisms with similar raw mutation rates). These differences can lead to different rates of neutral evolution and thus different rates for the molecular clock.

Finally, this activity can also be extended beyond a 50-minute period if the instructor builds in additional formative assessments (i.e., assessments designed to measure student learning and provide feedback to students on their own learning during the act of learning), which can also be deployed after class as homework or practice problems, or add additional areas of discussion. For instance, instructors can ask follow-up problems with different population sizes, mutation rates, and percentage of neutral mutations (versus those that are under purifying selection) to provide additional practice and check if students are understanding the concepts from the activity. Instructors can also vary the ploidy of the organism in the population, which would not impact the rate of neutral evolution; the rate of neutral evolution would still be equal to the rate of neutral mutation. The number of mutations that arise each generation would change (e.g., a diploid organism would have more mutations arise each generation than in a haploid organism if they both have identical rates of mutation, as measured by the number of mutations per allele). In addition, the probability of one of those mutations reaching fixation would be different given that the number of alleles in the gene pool would vary depending on ploidy (e.g., a mutation in a population of 100 haploid individuals would have a 1/100 chance of reaching fixation, while this would be 1/200 for a population of 100 diploid individuals). However, the total number of mutations expected to reach fixation due to drift would be equivalent between these haploid and diploid populations if the rates of neutral mutation were identical, independent of population size. Instructors may also wish to discuss the continuing scientific discourse over the significance of the neutral theory of molecular evolution (e.g., see Jensen et al., 2019; Kern & Hahn, 2018) and highlight how this reflects the nature of science.

*Other resources*

In addition to the resources cited above, instructors may find it helpful to consult additional texts that provide additional details on the neutral theory, including *Elements of Evolutionary Genetics* by Brian and Deborah Charlesworth (Charlesworth & Charlesworth, 2010) and *Evolution and Selection of Quantitative Traits* by Bruce Walsh and Michael Lynch (Walsh & Lynch, 2018). There are also several resources to help guide the teaching of the molecular clock, which is not the focus of this activity. Instructors may wish to refer to the supplemental table included in this paper to locate relevant papers for teaching molecular clock, which includes several papers that present activities or curriculum to introduce key concepts relating to molecular clock (e.g., Babaian & Kumar, 2020; Maroja & Wilder, 2012; Westerling, 2008; Yamanoi, Takemura, Sakura, & Kazama, 2012).

*General recommendations for teaching molecular evolution*

We conclude our instructor guide by providing several general recommendations for instructors wishing to teach molecular evolution in undergraduate biology. While these suggestions are broadly applicable and are not specific to just the teaching of the neutral theory, we include them here in the context of molecular evolution to better support instructors’ development of activities relating to molecular evolution.

1. **Integrate the teaching of *Vision & Change* core competencies.** We encourage instructors who are teaching molecular evolution to integrate core concepts highlighted in the *Vision & Change* report (American Association for the Advancement of Science (AAAS), 2009), and note how several sub-disciplines of molecular evolution may offer logical opportunities for doing so. For instance, one of the core concepts is quantitative reasoning. Our highlighted activity provides a unique chance to advance quantitative reasoning skills, with students mathematically calculating rates of change and accumulated mutations each generation and interpreting the biological significance of their calculations. Similarly, instructors may wish to highlight the process of science as they explain the relevance of certain areas of molecular evolution. For instance, our understanding of protein evolution has changed over time with new scientific advances and data challenging past assumptions and inferences (Pál, Papp, & Lercher, 2006). Instructors may wish to have students read and review such historical and new studies, which advances students’ knowledge of the process of science as well as develops students’ ability to design and evaluate scientific studies. We urge instructors to review these core competencies and consider ways to integrate these skills into the teaching of molecular evolution. Instructors may wish to review the Bioskills Guide, which provides more details on these core competencies and a suggested list of course-learning outcomes aligned with each core competency (Clemmons, Timbrook, Herron, & Crowe, 2020).
2. **Follow principles of backward design.** We have followed the principles of backward design each time we have taught molecular evolution. Backward design is the principle that instructors should begin by identifying a set of learning goals and objectives, then write assessments aligned with such learning goals, prior to designing the activities to support students mastering those learning goals (Reynolds & Kearns, 2017; Roth, 2007). Given that there are no set published standards for learning goals in molecular evolution, it is imperative that instructors start by considering what learning goals and specific learning objectives they want students to master when learning about molecular evolution. Given the rapidly changing nature of the field, we encourage instructors to consult the literature for relevant reviews on different areas of molecular evolution to contextualize any recent advances in the field. For example, there are reviews for molecular population genetics and evolution (Casillas & Barbadilla, 2017), rates of mutation (Baer, Miyamoto, & Denver, 2007; Lynch et al., 2016), and chromatin evolution and epigenetics (Dai, Ramesh, & Locasale, 2020; Talbert, Meers, & Henikoff, 2019), and likely similar review papers for many other sub-disciplines within molecular evolution. This literature can be used to inform the key learning objectives, and instructors can then use these learning objectives to design assessments. Given that our literature review found that there are not any validated instruments for assessing student learning in molecular evolution, it is incumbent upon instructors to write formative and summative assessments that allow instructors to identify naïve conceptions and measure mastery of learning objectives. We encourage instructors to review the existing literature base for suggestions on writing assessments (e.g., Suskie, 2018).
3. **Consider introducing principles of molecular evolution in introductory biology courses.** Our work identified that there were very few published activities for teaching molecular evolution at the introductory biology level, potentially suggesting that molecular evolution is not frequently taught at this level. In addition, we note that molecular evolution has not been explicitly included in the list of topics derived from *Vision & Change* core concepts, again suggesting low levels of coverage in introductory biology courses (Brownell, Freeman, Wenderoth, & Crowe, 2014; Cary & Branchaw, 2017). However, we note that many principles from molecular evolution integrate multiple core concepts. For instance, examining changes in rates of mutation and its impact on protein function spans across the core concepts of evolution, information flow, and structure and function, allowing instructors to address three of the five core concepts through teaching one topic. We also found a significant number of published papers with curriculum on molecular evolution for high school students, suggesting that many principles of molecular evolution would be appropriate and accessible for introductory biology students. Thus, we encourage instructors of introductory biology to consider integrating more molecular evolution whenever possible.

***Student handout***

**Learning objectives:**

At the end of this activity, you should be able to:

- Mathematically calculate the rate of neutral evolution in different populations
- Explain why the rate of neutral evolution is equal to the rate of neutral mutation under the neutral theory
- Draw inferences about how a constant rate of neutral evolution can inform the molecular clock

Consider a population of 200 humans that you genotype for the alkaline phosphatase (*ALP*) gene. Suppose that this gene has two alleles, A1 and A2, as shown below:

*Allele A1:*

5’ ATGCAC**C**GATCATAA 3’

*Allele A2:*

5’ ATGCAC**G**GATCATAA 3’

1. You find that 20 of the humans are A1/A1, 80 are A1/A2, and the remainder are A2/A2. Assume that while mutations occur, they are so rare that you can ignore mutations for now. In addition, alleles A1 and A2 have equal fitness, and no migration is occurring. What is the probability of allele A1 reaching fixation? What about A2? Assume that these two alleles are evolving independently of each other.

Now suppose you have a rate of mutation of two mutations for every one hundred **alleles** per generation. Mutations are so rare and random that you can always assume that a mutation will create a new allele, and that you will never get the same mutation twice. For example, suppose this mutation creates allele A3:

*Allele A3:*

5’ ATGCAC**C**GAT**G**ATAA 3’

1. How many mutations would you expect to get in your population of 200 humans after one generation? Write down your allelic frequencies for any new alleles you have.
2. Now suppose that half of these new mutations are under purifying selection. Assuming that there is strong purifying selection, predict what would happen to these mutations over time.
3. Let’s assume that the remaining mutations are not under any selective pressure so thus are evolving neutrally. Given this, what is the rate of **neutral** mutation in this population?
4. Now, pick one of the neutral mutations. What is the probability of it reaching fixation in this population? Why are we ignoring the impacts of selection here?
5. Assume that the probability of a single mutation reaching fixation is the same as the frequency of that mutation in the population. Given this assumption, what is the total number of neutral mutations that will reach fixation in this population?
6. How does your answer in the previous question compare to the rate of neutral mutation?
7. Re-do questions 2-7 here, but instead of the rate of mutation being two new mutations for every hundred alleles per generation, change the rate of mutation to two new mutations for every 200 alleles. How do your answers change to each part? In particular, does your answer change to question 7?

Now, let’s consider a larger population of 6000 humans, but with the same rate of mutation as before of one mutation per 100 alleles per generation.

1. Assume that half of the mutations are under purifying selection. What is the rate of neutral mutation in this population?
2. How many new neutral mutations would you expect in one generation, still assuming that half of the mutations are under purifying selection? What is the rate of neutral mutation?
3. For each new neutral mutation, what is its probability of fixation?
4. What is the total number of neutral mutations that will reach fixation in this population?
5. How does your answer to the previous question compare to the rate of neutral mutation?
6. Re-do questions 9-13 here, but instead of the rate of mutation being two new mutations for every hundred alleles per generation, change the rate of mutation to two new mutations for every 600 alleles. How do your answers change to each part? In particular, does your answer change to question 13?
7. Reflect on your exploration. What inferences can you draw about the **rate of neutral mutations reaching fixation in a population if neutrality holds?**
8. Suppose you track this population of humans over 100 generations. Every 20 generations, you randomly sample a number of individuals, and track how many genetic differences there are from the original generation. Predict your results by drawing a graph below with your expected data. Be sure to label both axes. Assume that there are no changes in the rate of mutation over time.
9. Did you predict a linear or non-linear trend in the previous question? Explain your reasoning and connect this to the neutral theory.

***Instructor key***

1. You find that 20 of the humans are A1/A1, 80 are A1/A2, and the remainder are A2/A2. Assume that while mutations occur, they are so rare that you can ignore mutations for now. In addition, alleles A1 and A2 have equal fitness, and no migration is occurring. What is the probability of allele A1 reaching fixation? What about A2? Assume that these two alleles are evolving independently of each other.

Students should understand, prior to this activity, that the probability of a given allele reaching fixation is equal to its frequency if drift is the only force at play. Thus, students here should calculate the allelic frequencies of A1 and A2. Since there are 200 individuals, there must be 400 total alleles in the population since humans are diploid. Of these 400 alleles, there are 120 A1 alleles (40 from the homozygous A1/A1 individuals and 80 from the heterozygote). Thus, the allelic frequency of A1 is 0.3, which is equal to its probability of fixation. The allelic frequency of A2 (and its probability of fixation) must be 0.7 since these are the only two alleles in the population.

Now suppose you have a rate of mutation of two mutations for every one hundred **alleles** per generation. Mutations are so rare and random that you can always assume that a mutation will create a new allele, and that you will never get the same mutation twice. For example, suppose this mutation creates allele A3:

*Allele A3:*

5’ ATGCAC**C**GAT**G**ATAA 3’

1. How many mutations would you expect to get in your population of 200 humans after one generation? Write down your allelic frequencies for any new alleles you have.

We have 400 total alleles, and a mutation rate of 2 new mutations per 100 alleles per generation. Thus, we expect eight new alleles in one generation. Each new allele would have a frequency of 1/400 since each mutation only occurs once. Instructors may wish to introduce these new alleles as A3, A4, A5, A6, A7, A8, A9, and A10 to promote students conceptualizing that a change in DNA would lead to a new allele here.

1. Now suppose that half of these new mutations are under purifying selection. Assuming that there is strong purifying selection, predict what would happen to these mutations over time.

Purifying selection would likely remove these mutations from the population. While it is possible that drift can increase the frequency of deleterious alleles, purifying selection (particularly if it is strong) will lead to decreased frequencies of these alleles. Thus, instructors can describe these alleles as likely being transient in the population, i.e., they will occur and then likely be eliminated from the population.

1. Let’s assume that the remaining mutations are not under any selective pressure so thus are evolving neutrally. Given this, what is the rate of **neutral** mutation in this population?

We have eight new mutations in the population. If half are under purifying selection, then half are evolving neutrally. Thus, four new mutations in the population this generation will be evolving neutrally (out of 400 total alleles), or a neutral mutation rate of 4/400 = 1/100. Instructors can also indicate to students that the rate of all mutations (i.e., the raw mutation rate) was two mutations per 100 alleles per generation. Thus, assuming that half of all mutations are under purifying selection, this means the rate of neutral evolution is half of the raw mutation rate, or ½ * 2/100, which is 1/100.

1. Now, pick one of the neutral mutations. What is the probability of it reaching fixation in this population? Why are we ignoring the impacts of selection here?

The probability of any allele reaching fixation is equal to its frequency here, since we are assuming drift is the only force acting upon this allele. Thus, the probability of fixation must be 1/400. We are ignoring the impacts of selection given that these are neutral mutations, i.e., these mutations are not under any form of selection.

1. Assume that the probability of a single mutation reaching fixation is the same as the frequency of that mutation in the population. Given this assumption, what is the total number of neutral mutations that will reach fixation in this population?

Here, we have four new neutral mutations, each with a 1/400 probability of reaching fixation. Thus, each mutation will contribute 1/400 mutations eventually reaching fixation. The total number of neutral mutations that will reach fixation is thus 4 * 1/400, or 1/100.

1. How does your answer in the previous question compare to the rate of neutral mutation?

Students should recognize that the number of neutral mutations that will reach fixation is equal to the rate of neutral mutation (1/100 here). Thus, the rate of neutral evolution is equal to the rate of neutral mutation.

1. Re-do questions 2-7 here, but instead of the rate of mutation being two new mutations for every hundred alleles per generation, change the rate of mutation to two new mutations for every 200 alleles. How do your answers change to each part? In particular, does your answer change to question 7?

Students should calculate that with a lower rate of mutation, there are now fewer mutations (4 mutations in our population of 200 diploid individuals) occurring each generation. If we assume half are neutral mutations, this means that there are 2 new neutral mutations this generation and that the rate of neutral mutation is 2 neutral mutations over 400 alleles (or 1/200). The probability of a specific new mutation reaching fixation is still 1/400; thus, the number of new neutral mutations reaching fixation is 2 * 1/400, or 1/200. Instructors should guide students to recognize that this is still equivalent to the rate of neutral mutation.

Now, let’s consider a larger population of 6000 humans, but with the same rate of mutation as before of two mutations per 100 alleles per generation.

1. Assume that half of the mutations are under purifying selection. What is the rate of neutral mutation in this population?

If there are 6000 individuals, we now have 12,000 alleles. 12,000 alleles multiplied by 2 new mutations per 100 alleles leads to 240 new mutations.

1. How many new neutral mutations would you expect in one generation, still assuming that half of the mutations are under purifying selection? What is the rate of neutral mutation?

Half of the 240 mutations (thus, 120 mutations) would be evolving neutrally. The neutral mutation rate here is ½ * 2/100, or 1 new neutral mutation per 100 alleles.

1. For each new neutral mutation, what is its probability of fixation?

The probability is equal to its frequency in the population, so this would be 1/12,000.

1. What is the total number of neutral mutations that will reach fixation in this population?

There are 120 new neutral mutations, each with a 1/12,000 chance of reaching fixation. Thus, the number of mutations reaching fixation is 120 * 1/12,000, or 1/100.

1. How does your answer to the previous question compare to the rate of mutation?

Students should again recognize the pattern that the rate of neutral evolution is equivalent to the neutral mutation rate.

1. Re-do questions 9-13 here, but instead of the rate of mutation being two new mutations for every hundred alleles per generation, change the rate of mutation to two new mutations for every 600 alleles. How do your answers change to each part? In particular, does your answer change to question 13?

In this scenario, the mutation rate decreases (as does the rate of neutral evolution, which will be half the raw mutation rate if we assume that half the mutations will be neutral), as does the number of new neutral mutations each generation (20) and the rate of neutral evolution (1/600). The rate of neutral evolution is still equal to the rate of neutral mutation.

1. Reflect on your exploration. What inferences can you draw about the **rate of new mutations reaching fixation in a population if neutrality holds?**

Instructors should guide students to think critically about their results and recognize that the rate of new neutral mutations reaching fixation in a population is always equal to the rate of neutral mutation, independent of population size.

1. Suppose you track this population of humans over 100 generations. Every 20 generations, you randomly sample a number of individuals, and track how many genetic differences there are from the original generation. Predict your results by drawing a graph below with your expected data. Be sure to label both axes. Assume that there are no changes in the rate of mutation over time.

Students should draw a graph with time on the x-axis (in generations) and the amount of genetic differences on the y-axis, showing a comparison between the original sample and the new sample at a given time. Students should provide a linear trend for this line, starting at the origin. Instructors should note that the assumption of constant rates of mutation may not be true for many organisms, which may vary in organisms for a variety of reasons.

1. Did you predict a linear or non-linear trend in the previous question? Explain your reasoning and connect this to the neutral theory.

Students should have a line (a linear trend), given the constant accumulation of changes. The neutral theory predicts that the rate of neutral evolution is constant (and equal to the rate of neutral mutation), thus driving this constant, regular accumulation of neutral changes. This trend is known as the molecular clock.

***References***

Abraham, J. K., Meir, E., Perry, J., Herron, J. C., Maruca, S., & Stal, D. (2009). Addressing Undergraduate Student Misconceptions about Natural Selection with an Interactive Simulated Laboratory. *Evolution: Education and Outreach*, *2*(3), 393–404. doi: 10.1007/s12052-009-0142-3

American Association for the Advancement of Science (AAAS). (n.d.). Vision and Change in Undergraduate Biology Education: A View for the 21st Century. Retrieved February 25, 2022, from https://visionandchange.org/about-vc-a-call-to-action-2011/

Anderson, D. L., Fisher, K. M., & Norman, G. J. (2002). Development and evaluation of the conceptual inventory of natural selection. *Journal of Research in Science Teaching*, *39*(10), 952–978. doi: 10.1002/tea.10053

Andrews, T. M., Price, R. M., Mead, L. S., McElhinny, T. L., Thanukos, A., Perez, K. E., … Lemons, P. P. (2012). Biology Undergraduates’ Misconceptions about Genetic Drift. *CBE—Life Sciences Education*, *11*(3), 248–259. doi: 10.1187/cbe.11-12-0107

Andrews, Tessa M., Kalinowski, S. T., & Leonard, M. J. (2011). “Are Humans Evolving?” A Classroom Discussion to Change Student Misconceptions Regarding Natural Selection. *Evolution: Education and Outreach*, *4*(3), 456–466. doi: 10.1007/s12052-011-0343-4

Babaian, C., & Kumar, S. (2020). Molecular Memories of a Cambrian Fossil. *The American Biology Teacher*, *82*(9), 586–595. doi: 10.1525/abt.2020.82.9.586

Baer, C. F., Miyamoto, M. M., & Denver, D. R. (2007). Mutation rate variation in multicellular eukaryotes: Causes and consequences. *Nature Reviews Genetics*, *8*(8), 619–631. doi: 10.1038/nrg2158

Baumgartner, E., & Duncan, K. (2009). Evolution of Students’ Ideas about Natural Selection through a Constructivist Framework. *The American Biology Teacher*, *71*(4), 218–227. doi: 10.2307/27669415

Beggrow, E. P., & Nehm, R. H. (2012). Students’ Mental Models of Evolutionary Causation: Natural Selection and Genetic Drift. *Evolution: Education and Outreach*, *5*(3), 429–444. doi: 10.1007/s12052-012-0432-z

Brownell, S. E., Freeman, S., Wenderoth, M. P., & Crowe, A. J. (2014). BioCore Guide: A Tool for Interpreting the Core Concepts of Vision and Change for Biology Majors. *CBE—Life Sciences Education*, *13*(2), 200–211. doi: 10.1187/cbe.13-12-0233

Cary, T., & Branchaw, J. (2017). Conceptual Elements: A Detailed Framework to Support and Assess Student Learning of Biology Core Concepts. *CBE—Life Sciences Education*, *16*(2), ar24. doi: 10.1187/cbe.16-10-0300

Casillas, S., & Barbadilla, A. (2017). Molecular Population Genetics. *Genetics*, *205*(3), 1003–1035. doi: 10.1534/genetics.116.196493

Charlesworth, B., & Charlesworth, D. (2010). *Elements of Evolutionary Genetics*. Roberts and Company. Retrieved from https://www.research.ed.ac.uk/en/publications/elements-of-evolutionary-genetics

Clemmons, A. W., Timbrook, J., Herron, J. C., & Crowe, A. J. (2020). BioSkills Guide: Development and National Validation of a Tool for Interpreting the Vision and Change Core Competencies. *CBE—Life Sciences Education*, *19*(4), ar53. doi: 10.1187/cbe.19-11-0259

Dai, Z., Ramesh, V., & Locasale, J. W. (2020). The evolving metabolic landscape of chromatin biology and epigenetics. *Nature Reviews Genetics*, *21*(12), 737–753. doi: 10.1038/s41576-020-0270-8

Ferrari, M., & Chi, M. T. H. (1998). The nature of naive explanations of natural selection. *International Journal of Science Education*, *20*(10), 1231–1256. doi: 10.1080/0950069980201005

Furrow, R. E., & Hsu, J. L. (2019). Concept inventories as a resource for teaching evolution. *Evolution: Education and Outreach*, *12*(1), 2. doi: 10.1186/s12052-018-0092-8

Greene Jr., E. D. (1990). The logic of university students’ misunderstanding of natural selection. *Journal of Research in Science Teaching*, *27*(9), 875–885. doi: 10.1002/tea.3660270907

Gregory, T. R. (2009). Understanding Natural Selection: Essential Concepts and Common Misconceptions. *Evolution: Education and Outreach*, *2*(2), 156–175. doi: 10.1007/s12052-009-0128-1

Hsu, J., Imad, M., & Wilson*, K. M. (2021). *Furry with a chance of evolution: Exploring genetic drift with tuco-tucos*. doi: 10.24918/cs.2019.17

Jensen, J. D., Payseur, B. A., Stephan, W., Aquadro, C. F., Lynch, M., Charlesworth, D., & Charlesworth, B. (2019). The importance of the Neutral Theory in 1968 and 50 years on: A response to Kern and Hahn 2018. *Evolution*, *73*(1), 111–114. doi: 10.1111/evo.13650

Kalinowski, S. T., Andrews, T. M., Leonard, M. J., & Snodgrass, M. (2012). Are Africans, Europeans, and Asians Different “Races”? A Guided-Inquiry Lab for Introducing Undergraduate Students to Genetic Diversity and Preparing Them to Study Natural Selection. *CBE—Life Sciences Education*, *11*(2), 142–151. doi: 10.1187/cbe.11-09-0087

Kalinowski, S. T., Leonard, M. J., & Taper, M. L. (2016). Development and Validation of the Conceptual Assessment of Natural Selection (CANS). *CBE—Life Sciences Education*, *15*(4), ar64. doi: 10.1187/cbe.15-06-0134

Kern, A. D., & Hahn, M. W. (2018). The Neutral Theory in Light of Natural Selection. *Molecular Biology and Evolution*, *35*(6), 1366–1371. doi: 10.1093/molbev/msy092

Lawson, A. E., & Thompson, L. D. (1988). Formal reasoning ability and misconceptions concerning genetics and natural selection. *Journal of Research in Science Teaching*, *25*(9), 733–746. doi: 10.1002/tea.3660250904

Lynch, M., Ackerman, M. S., Gout, J.-F., Long, H., Sung, W., Thomas, W. K., & Foster, P. L. (2016). Genetic drift, selection and the evolution of the mutation rate. *Nature Reviews Genetics*, *17*(11), 704–714. doi: 10.1038/nrg.2016.104

Maroja, L. S., & Wilder, J. A. (2012). Where Do I Come From? Using Student’s Mitochondrial DNA to Teach About Phylogeny, Molecular Clocks, and Population Genetics. *Evolution: Education and Outreach*, *5*(3), 501–507. doi: 10.1007/s12052-012-0436-8

Nehm, R. H., Beggrow, E. P., Opfer, J. E., & Ha, M. (2012). Reasoning About Natural Selection: Diagnosing Contextual Competency Using the ACORNS Instrument. *The American Biology Teacher*, *74*(2), 92–98. doi: 10.1525/abt.2012.74.2.6

Nehm, R. H., & Reilly, L. (2007). Biology Majors’ Knowledge and Misconceptions of Natural Selection. *BioScience*, *57*(3), 263–272. doi: 10.1641/B570311

Nehm, R. H., & Schonfeld, I. S. (2008). Measuring knowledge of natural selection: A comparison of the CINS, an open-response instrument, and an oral interview. *Journal of Research in Science Teaching*, *45*(10), 1131–1160. doi: 10.1002/tea.20251

Pál, C., Papp, B., & Lercher, M. J. (2006). An integrated view of protein evolution. *Nature Reviews Genetics*, *7*(5), 337–348. doi: 10.1038/nrg1838

Price, R. M., Andrews, T. C., McElhinny, T. L., Mead, L. S., Abraham, J. K., Thanukos, A., & Perez, K. E. (2014). The Genetic Drift Inventory: A Tool for Measuring What Advanced Undergraduates Have Mastered about Genetic Drift. *CBE—Life Sciences Education*, *13*(1), 65–75. doi: 10.1187/cbe.13-08-0159

Reynolds, H. L., & Kearns, K. D. (2017). A Planning Tool for Incorporating Backward Design, Active Learning, and Authentic Assessment in the College Classroom. *College Teaching*, *65*(1), 17–27. doi: 10.1080/87567555.2016.1222575

Roth, D. (2007). Understanding by Design: A Framework for Effecting Curricular Development and Assessment. *CBE—Life Sciences Education*, *6*(2), 95–97. doi: 10.1187/cbe.07-03-0012

Russo, C. A. M., & Voloch, C. M. (2012). Beads and Dice in a Genetic Drift Exercise. *Evolution: Education and Outreach*, *5*(3), 494–500. doi: 10.1007/s12052-012-0438-6

Settlage Jr., J. (1994). Conceptions of natural selection: A snapshot of the sense-making process. *Journal of Research in Science Teaching*, *31*(5), 449–457. doi: 10.1002/tea.3660310503

Smith, M. K., Wood, W. B., & Knight, J. K. (2008). The Genetics Concept Assessment: A New Concept Inventory for Gauging Student Understanding of Genetics. *CBE—Life Sciences Education*, *7*(4), 422–430. doi: 10.1187/cbe.08-08-0045

Suskie, L. (2018). *Assessing student learning: A common sense guide*. John Wiley & Sons.

Talbert, P. B., Meers, M. P., & Henikoff, S. (2019). Old cogs, new tricks: The evolution of gene expression in a chromatin context. *Nature Reviews Genetics*, *20*(5), 283–297. doi: 10.1038/s41576-019-0105-7

Tibell, L. A. E., & Harms, U. (2017). Biological Principles and Threshold Concepts for Understanding Natural Selection. *Science & Education*, *26*(7), 953–973. doi: 10.1007/s11191-017-9935-x

Walsh, B., & Lynch, M. (2018). *Evolution and Selection of Quantitative Traits*. Oxford University Press.

Westerling, K. E. (2008). Using Playing Cards To Simulate a Molecular Clock. *The American Biology Teacher*, *70*(8). doi: 10.1662/0002-7685(2008)70[37:UPCTSA]2.0.CO;2

Yamanoi, T., Takemura, M., Sakura, O., & Kazama, T. (2012). Development and Evaluation of an Activity to Teach Molecular Phylogeny,Deep Time and Classification Systems for Japanese High School Students. *The Asian Journal of Biology Education*, *6*, 13–25. doi: 10.57443/ajbe.6.0_13

Zhao, F., & Schuchardt, A. (2019). Exploring Students’ Descriptions of Mutation from a Cognitive Perspective Suggests How to Modify Instructional Approaches. *CBE—Life Sciences Education*, *18*(3), ar45. doi: 10.1187/cbe.18-11-0225

Ziadie, M. A., & Andrews, T. C. (2018). Moving Evolution Education Forward: A Systematic Analysis of Literature to Identify Gaps in Collective Knowledge for Teaching. *CBE—Life Sciences Education*, *17*(1), ar11. doi: 10.1187/cbe.17-08-0190
